# Impregnated Polymeric Sorbent for the Removal of Noble Metal Ions from Model Chloride Solutions and the RAM Module

**DOI:** 10.3390/ma17061234

**Published:** 2024-03-07

**Authors:** Karolina Zinkowska, Zbigniew Hubicki, Grzegorz Wójcik

**Affiliations:** Department of Inorganic Chemistry, Institute of Chemical Sciences, Faculty of Chemistry, Maria Curie-Skłodowska University, Maria Curie-Skłodowska Sq. 2, 20-031 Lublin, Poland; grzegorz.wojcik2@mail.umcs.pl

**Keywords:** noble metals, gold, e-waste, ion exchange, impregnated resin, sorption

## Abstract

Nowadays, there is a need for new sources of noble metals due to their dwindling natural resources. This paper presents studies on the sorption of noble metals such as Au(III), Pt(IV), Pd(II) and Rh(III) from model chloride solutions on a newly prepared Amberlite XAD-16–Aliquat 336 sorbent. A “warm impregnation” method without the use of toxic organic solvents was applied to impregnate the polymer matrix. The influence of such factors as hydrochloric acid concentration, sorbent mass and phase contact time was investigated. Kinetic as well as adsorption isotherm studies were carried out. The sorption capacity of the synthesized sorbent was Au(III)—94.34 mg/g, Pt(IV)—45.35 mg/g and Pd(II)—46.03 mg/g. Based on thermodynamic considerations, their sorption proved to be endothermic, as the values of ΔH° > 0. Sorption was spontaneous and favourable (ΔG° < 0). After leaching the RAM module, there was obtained a real solution, in which the metal contents were determined: 38.10 mg/g of gold and 1.76 mg/g of palladium. Totals of 99.9% of gold and 45.4% of palladium were removed from the real leaching solution, with other elements in the solution.

## 1. Introduction

Noble metals, particularly gold, platinum, palladium and rhodium, are applied in various industries, from jewelry to medicine. The demand for these metals is predicted to increase in the electrical and electronic industries [1]. In the manufacture of mobile phones, computers, TV sets, etc., precious metals are used to coat contacts, pins, chips and electrodes. Of all noble metals, gold is characterized by the best properties such as corrosion and acid resistance as well as good electrical conductivity. Therefore, it is the most commonly chosen metal for such applications [2].

Such a large and ever-growing demand for noble metals is due to constant technological advances. For this reason, and due to the fact that they are exhaustible raw materials, their resources are drastically decreasing year by year. New materials with the same or even better properties than those of noble metals are being sought; however, currently, their replacing is impossible. In addition, extraction of noble metals from natural deposits (e.g., extraction of gold or platinum) is associated with huge energy expenditures, which affect the environment negatively [3]. Therefore, the recovery of noble metals from scraps, primarily from waste electrical and electronic equipment (WEEE), is of significant importance. E-waste includes various metals (e.g., noble metals), plastics, as well as glass and ceramic materials. Their composition depends on type as well as the manufacturers. The literature reports that a ton of e-waste can contain approximately 10–1000 g of gold [4]. Approximately 320 ppm of gold, 3000 ppm of silver and 120 ppm of palladium can be found in a cell phone. However, in a printed circuit board from a computer, there is approximately 200 ppm of gold, 900 ppm of silver and 80 ppm of palladium [5].

The European Union was the first to issue a comprehensive piece of legislation on WEEE management. Directive 2003/108/EC, which amends Directive 2002/96/EC on waste electrical and electronic equipment (WEEE), also required producers of such devices to manage the waste generated from them. At the same time, according to the directive, a number of different substances used in the production of EEE were banned due to harmful waste after the process [6]. Later, more directives were formed, and their regulations became more and more restrictive [7]. A total of 193 UN member states have the 2030 Agenda, aimed at achieving specific tasks by 2030. In March 2015, there were introduced 330 indicators for monitoring sustainability. The 12th of 17 goals refers to ensuring sustainable consumption and production patterns, for instance, minimizing chemical pollution, sound waste management, but most importantly preventing waste through its treatment and recycling [8].

According to Eurostat statistics, in 2012 there were approximately 7.3 million tons of electrical and electronic equipment on the European Union market, of which only 3 million tons were collected, while 3.1 million tons were processed and 2.4 million tons were recycled. In 2021, the amount of equipment on the market increased to 13.5 million tons, those processed to 4.8 million tons and recycled to 4 million tons [9]. The statistics show how huge amounts of WEEE are not recycled, and these are the data only for Europe. Processing such enormous amounts of e-waste will increase the supply of noble metals for reuse significantly.

Noble metal recovery is mainly based on pyrometallurgical processes, which involve the smelting of electronic and electrical scraps in high-temperature furnaces and using hydrometallurgical processes with the use of various chemical reagents to extract desired metals [10]. Pyrolysis furnaces maintain temperatures, which are higher than the melting point of the main metals (Pb, Zn, Cd, Cu, Sb) contained in WEEE. Therefore, they can be easily separated from the remaining metallic fraction, with some noble metals. However, at the same time, this can cause losses of the recovered metals due to their combustion [11]. J. Willner et al. placed 100 g samples obtained from the circuit boards as well as the spent car exhaust converters in the electric inductive furnace at 1700 °C for 1 h. The studies proved that average recovery degrees of the metals were 78.3% of Pt, 81.2% Cu, 83.7% of Au and 60.6% of Fe by the pyrometallurgical method [12]. Hydrometallurgical processes have the advantage over pyrometallurgical ones by requiring small energy and simple equipment as well as being inexpensive. The disadvantage of this method is the large amount of generated chemical waste. Gold and silver leaching can be performed by cyanidation. P. M. Petter et al. treated 10 g of PCB sample with commercial potassium cyanide-based reagent for 2–4 h. After leaching, the extraction degree of gold and silver amounted to approximately 60–70% [13]. Another reagent for leaching can be thiourea. It is less toxic than cyanide and has better kinetics. With a 24 g/L concentration of thiourea, 0.6% of iron ions and particle size of 100 mesh at a temperature of 25 °C, there can be obtained the extraction degrees of 90% Au and 50% Ag [14]. Separation of noble metals from the solutions after leaching is made by means of adsorption, ion exchange or solvent extraction [15].

Random access memory (RAM) modules are used to store data on computers and contain, among other things, some amounts of noble metals, mainly gold and silver. This is a type of printed circuit board (PCBs). Some papers describe the recovery of noble metals from the RAM module. M. Tatariants et al. analyzed RAM contacts, showing that nickel and gold are the main components. First, plastic was separated from the metal parts by dissolving the brominated epoxy resin using dimethylacetamide. The metal parts were treated with nitric(V) acid, which dissolved nickel and copper, but not gold. Studies revealed that approximately 28 mg of gold was recovered from a single RAM module [16]. Mudila et al. carried out a study on the recovery of gold from PCBs derived from worn-out cell phones. Preliminary studies proved that powder obtained from the spent PCB contained 0.0013% wt. of gold. At first, pre-treatment using DMA (N,N—dimethylacetamide) delaminated PCB. Concentration of gold in the delaminated metal fraction was 0.046% wt. Dissolution of copper and other metals was performed using nitric acid(V). For gold leaching, PCB powder was treated with 3 M sulfuric acid and 3 M NaBr. Gold recovery was due to solvent extraction using primary, secondary, tertiary amide—R_3_NC(O)CH_2_C(Me)CH_2_CMe_3_ (1◦, R_1_ = R_2_ = H; 2◦, R_1_ = Me, R_2_ = H; 3◦, R_1_ = R_2_ = Me) and toluene. The best efficiency (over 99%) was obtained for tertiary amide [17]. Barnwal and Dhawan studied the recovery of gold and copper from discarded mobile printed circuit board. A 2 M solution of HCl with H_2_O_2_ as an oxidizing agent and solution of HNO_3_ was used. They mixed HCl and HNO_3_ at the ratio of 1:3 to make aqua regia since gold passing into solutions of individual acids was very slow. Urea was added to neutralize the acid. Sodium bisulfite was added to precipitate the gold. A total of 73% gold extraction was obtained [18]. Gámez et al. performed gold leaching with ammonia thiosulfate solutions. The concentration of gold in the spent circuit board was 453.4 mg/kg. The leaching efficiency using sodium thiosulfate was 81%. To remove gold ions from ammonia thiosulfate leachates, there was used the ion exchange resin MTA 5011 yielding 87% gold recovery [19].

In our study, solvent impregnated resin (SIR) was used. Solvent impregnated resins are great alternatives for extracting noble metals from waste solutions [20]. Their use minimizes chemical waste generated in solvent extraction, where large quantities of chemicals, often toxic organic solvents, are used. Moreover, they can be used for very low concentrations of elements and there are no problems with phase separation [21]. The XAD-16 sorbent was impregnated by means of a new method called ‘warm impregnation’ using the quaternary ammonium salt Aliquat 336. In our previous studies, SIRs for the sorption of noble metals, including gold, palladium and platinum, were applied giving positive results. Properties of commercially available adsorbents were altered by their impregnating with the extractant selective for noble metal ions [22,23]. The paper aimed at the sorption of Au(III), Pd(II), Pt(IV) and Rh(III) ions from chloride model solutions and the verification of suitability of the impregnated resin for the recovery of noble metals from the real system, i.e., digested random access memory module.

## 2. Materials and Methods

### 2.1. Materials

**Figure 1:**
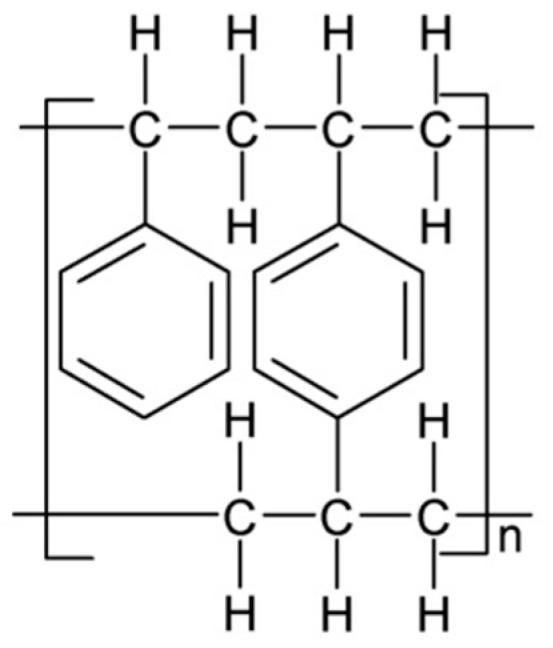
Structure of Amberlite XAD-16.

**Figure 2:**
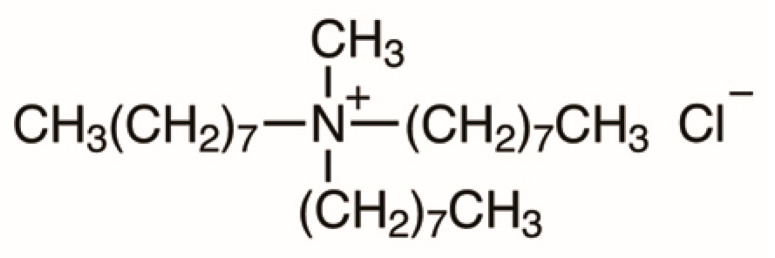
Structure of Aliquat 336.

Amberlite XAD-16 (Rohm and Haas, Esslingen, Germany) is a mesoporous, hydrophobic, nonionic, crosslinked polymer. Its structure is presented in Figure 1. It has a large specific surface area of 800 m^2^/g and pore size of 200 Å. Its form is made up of white translucent beads. The maximum temperature limit for this adsorbent is 150 °C. Due to its large specific surface area, pore size and great stability, it can be easily modified with various types of extractants [24]. The structure of Amberlite XAD-16 is provided in Figure 1. For the impregnation process, Aliquat 336 (Sigma Aldrich, Darmstadt, Germany) was used. This is a water insoluble quaternary ammonium salt (trioctylmethylammonium chloride, C_25_H_54_NCl) in the form of a yellowish viscous liquid. It is applied for some metals removal for water treatment and metal recovery. The structure of Aliquat 336 is presented in Figure 2.

Distilled water was obtained from Polwater DL2–150 system (Cracow, Poland).

Noble metal solutions were prepared from the HAuCl_4_, H_2_PtCl_6_ and H_2_PdCl_4_ solutions (POCH Gliwice, Gliwice, Poland). Reference solutions containing 1000 mg/L of each element were used to prepare standard solutions for the sorption studies. They contained 10 ppm of gold(III), platinum(IV), palladium(II) and rhodium(III).

### 2.2. Methods

#### 2.2.1. Impregnation Process

“Warm impregnation” is based on direct mixing of the sorbent and the extractant at an increased temperature. This method neglects the use of toxic organic solvents, which makes it more environmentally friendly. Amberlite XAD-16 (Rohm and Haas, Esslingen, Germany) was impregnated with Aliquat 336 (Sigma Aldrich, Darmstadt, Germany) at the weight ratio 1:1 at 333 K. After a few minutes of stirring, the impregnated sorbent became loose, so the beads did not stick together. This indicates that the entire volume of extractant was absorbed by the sorbent. This is most likely due to the large specific surface area of Amberlite XAD-16. This is an innovative impregnation method because it does not use toxic solvents such as acetone or chloroform. It proves to be simple and reproducible.

#### 2.2.2. Analysis of the SIR

A scanning electron microscopy (Phenom World scanning electron microscope, Thermo Scientific, Waltham, MA, USA), Fourier transform infrared spectroscopy–attenuated total reflectance (Agilent Cary 630, Agilent Technologies, Santa Clara, CA, USA) and low-temperature nitrogen adsorption measurements using an accelerated surface area and porosimetry system (analyzer ASAP 2045, Micromeritics, Norcross, GA, USA) were applied to examine and characterize the sorbent.

#### 2.2.3. Sorption and Desorption Studies

In order to characterize the sorption of Au(III), Pd(II), Pt(IV) and Rh(III) on impregnated resin Amberlite XAD-16–Aliquat 336, the effects of contact time, acid concentration and mass of sorbent were studied.

To study the effect of sorbent mass on noble metal ions’ sorption, samples with different masses of the sorbent (0.025, 0.05, 0.1, 0.15, 0.20, 0.25 g) were prepared. Samples were treated with 25 mL noble metal solution (10 ppm Au(III), Pd(II), Pt(IV) and Rh(III); 0.1 M hydrochloric acid). The samples were shaken in the laboratory shaker (Elpin + type 358, Elpin, Warsaw, Poland) and, after 24 h, the concentration of the noble metals was examined by inductively coupled plasma–optical emission spectrometry (Varian 720 ES ICP-OES, Richmond Scientific, Chorley, UK).

After the sorption studies, phases of samples were separated. Amberlite XAD-16–Aliquat 336 with adsorbed noble metal ions was treated with 25 mL desorbing solution (0.1–1 M thiourea in 0.1–1 M hydrochloric acid). Concentrations of desorbed metals ions were measured by inductively coupled plasma–optical emission spectrometry (Varian 720 ES ICP-OES).

The spectral lines using ICP-OES were 242.794 nm for Au(III); 340.458 nm for Pd(II); 214.424 nm for Pt(IV); 343.488 nm for Rh(III).

#### 2.2.4. Kinetic, Thermodynamic and Isotherm Studies

For sorption kinetic studies, the samples with 0.2 g of Amberlite XAD-16–Aliquat 336 were treated with 50 mL of noble metal solutions (10 ppm Au(III), Pd(II), Pt(IV) and Rh(III)) at hydrochloric acid concentration: 0.1 M; 1 M; 3 M; 6 M). The samples were shaken in the laboratory shaker (Elpin + type 358, Elpin, Poland) and, after a specific time (1; 5; 15; 30; 60; 120; 240; 360; 1440 min), the concentration of the noble metals was examined by inductively coupled plasma–optical emission spectrometry (Varian 720 ES ICP-OES).

To examine the sorption thermodynamics, 0.1 g of Amberlite XAD-16–Aliquat 336 was equilibrated with 25 mL noble metal solution (10 ppm Au(III), Pd(II), Pt(IV) and Rh(III)) in 0.1 M hydrochloric acid. The samples were shaken in a shaker bath at 293, 303, 333 K and, after 1440 min, noble metal concentrations were measured by inductively coupled plasma–optical emission spectrometry (Varian 720 ES ICP-OES).

To study sorption isotherms, 0.1 g of the impregnated sorbent was contacted with 25 mL of individual noble metal solutions (10, 100, 250, 500 ppm; 0.1 M hydrochloric acid). The samples were shaken in the laboratory shaker (Elpin + type 358, Elpin, Poland) and, after 1440 min, the concentration of noble metals was measured by inductively coupled plasma–optical emission spectrometry (Varian 720 ES ICP-OES).

#### 2.2.5. Noble Metal Recovery from the Spent RAM Module

The spent RAM module was leached to transfer noble metals into the solution. The HCl/H_2_O_2_ system was used for leaching. The single RAM module was contacted with 50 mL of 36% hydrochloric acid and the sample was heated at 333 K. A total of 50 mL of 30% hydrogen peroxide was added to the sample in portions. The mixture was heated for 3 h. Then, the mixture was filtered from e-waste residue. The concentration of noble metals was measured by inductively coupled plasma–optical emission spectrometry (Varian 720 ES ICP-OES).

A total of 0.1 g of Amberlite XAD-16–Aliquat 336 was treated with real leach solution containing noble metals. Concentrations of these metals were measured by inductively coupled plasma–optical emission spectrometry (Varian 720 ES ICP-OES).

## 3. Results and Discussion

### 3.1. Analysis of Impregnated XAD-16

**Figure 3:**
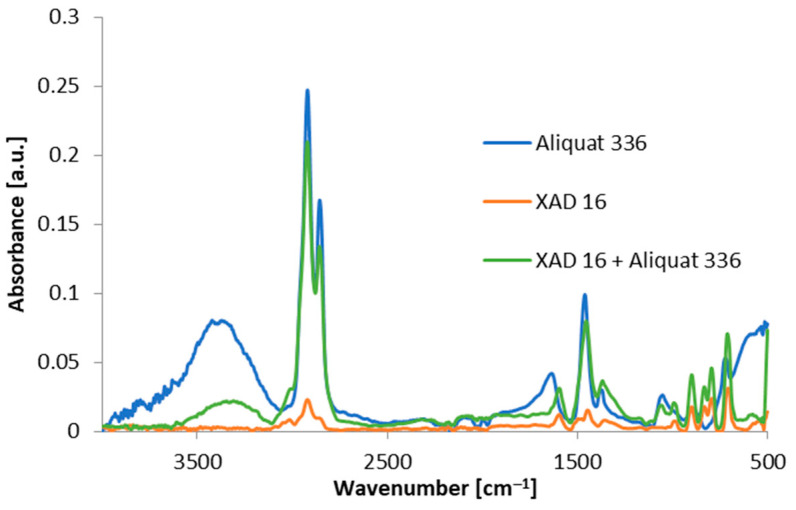
FTIR-ATR spectra of pure sorbent Amberlite XAD-16, pure Aliquat 336 and impregnated sorbent Amberlite XAD-16–Aliquat 336.

The FTIR-ATR spectra are presented in Figure 3. They refer to the impregnated sorbent as well as to separate ones: pure sorbent and extractant.

In the FTIR-ATR spectrum of Amberlite XAD-16 occurs bands of Aliquat 336 which show that the extractant has been loaded on the polymeric sorbent. It can be seen that, characteristic for Aliquat 336, bands of -CH_2_- and -CH_3_ groups corresponding to the asymmetric and symmetric stretching vibrations, 2921 cm^−1^ and 2865 cm^−1^, appear in the spectrum of impregnated resin. Moreover, the distinctive band involving quaternary ammonium salt, 1470 cm^−1^, is present in the spectrum. The aromatic stretches of -C-H- groups in pure Amberlite XAD-16, 3016 cm^−1^, were also found. Two bands of Amberlite XAD-16, 2921 cm^−1^ and 2864 cm^−1^, are responsible for asymmetric and symmetric stretching vibrations of -CH_2_- and -CH_3_.

**Figure 4:**
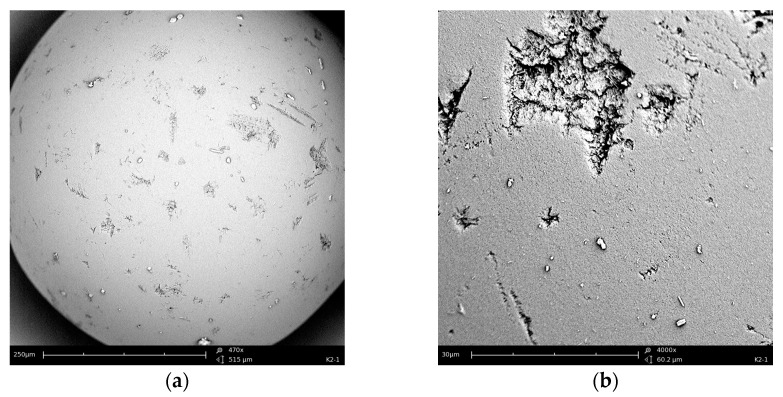

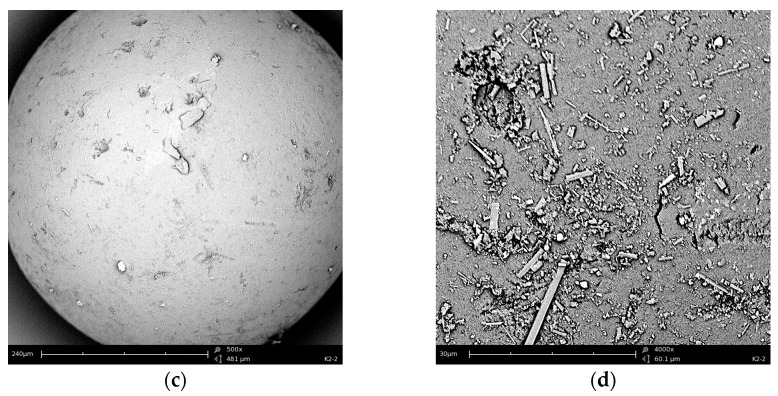
SEM pictures of Amberlite XAD-16: (**a**) Before impregnation mag. 500×; (**b**) Before impregnation mag. 4000×; (**c**) After impregnation with Aliquat 336 mag. 500×; (**d**) After impregnation with Aliquat 336 mag. 4000×.

The SEM pictures of the sorbent Amberlite XAD-16 before and after impregnation are presented in Figure 4. Figure 4a,b show pure sorbent Amberlite XAD-16 without the extractant; Figure 4c,d present sorbent Amberlite XAD-16 impregnated with Aliquat 336. After impregnation, only small amounts of Aliquat 336 can be seen on the sorbent surface. Due to the large specific surface area of Amberlite XAD-16, practically the entire volume of Aliquat 336 is absorbed into the pores.

**Figure 5:**
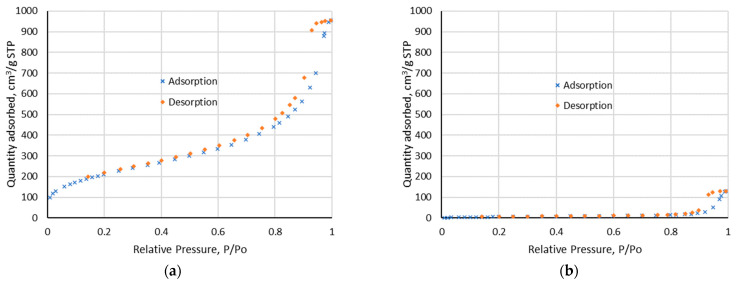
Isotherm linear plot: (**a**) before impregnation; (**b**) after impregnation.

Low-temperature (77 K) nitrogen adsorption measurements determined the specific surface area, total pore volume and pore size distribution. The nitrogen isotherms before and after impregnation process are presented in Figure 5. BJH measurements show a wide pore size distribution: 1.7–300 nm. Before impregnation, the BET surface area was 777.098 m^2^/g and after impregnation it decreased to 20.395 m^2^/g. The same can be observed for pore volume, which decreased from 1.475 cm^3^/g to 0.201 cm^3^/g, indicating that Aliquat 336 fills in pores of Amberlite XAD-16. As follows from the research, impregnation actually took place.

All methods, FTIR-ATR, SEM and ASAP, prove that impregnation of Amberlite XAD-16 by Aliquat 336 takes place.

### 3.2. Sorption Studies

#### 3.2.1. Influence of the Impregnated Sorbent Mass

**Figure 6:**
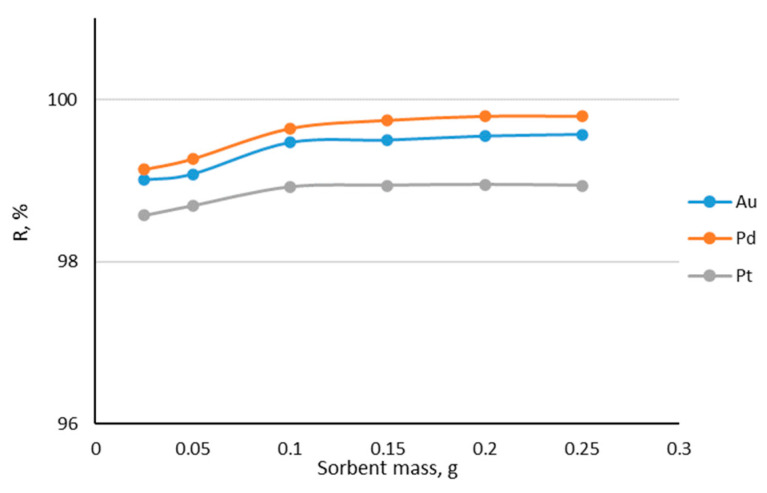
Dependence of %R on Amberlite XAD-16–Aliquat 336 mass (sorbent mass: 0.025; 0.05 0.1; 0.15; 0.2; 0.25 g; each metal concentration: 10 ppm; solution volume: 25 mL; HCl concentration: 0.1 M).

The effect of Amberlite XAD-16–Aliquat 336 mass on the noble metal ions sorption efficiency was investigated to find the optimal sorbent mass. Based on the graph in Figure 6, it can be concluded that increasing the mass of sorbent for the sorption of noble metal ions results in an increase in their removal percentage from the chloride model solution. However, at a sorbent mass of 0.1 g, further increasing the sorbent mass does not change the recovery percentage significantly. This indicates that this is the most optimal sorbent mass from which samples were prepared for further research.

#### 3.2.2. Influence of Hydrochloric Acid Concentration

**Figure 7:**
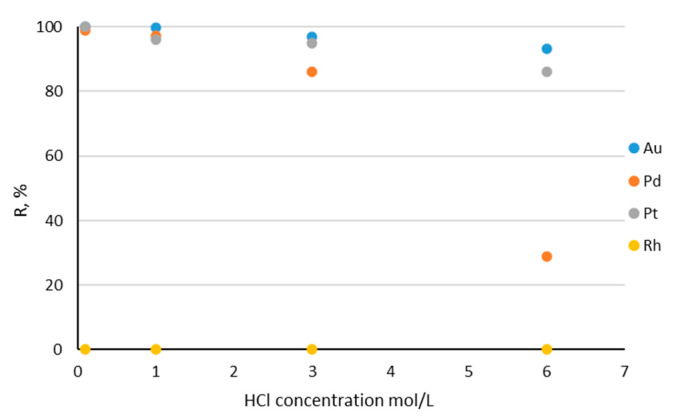
Dependence of %R on the hydrochloric acid concentrations (0.1; 1; 3; 6 M) for Au(III), Pd(II), Pt(IV) and Rh(III) (each metal concentration: 10 ppm; sorbent mass: 0.1 g; solution volume: 25 mL).

The influence of hydrochloric acid concentration on the investigated noble metal ions sorption on Amberlite XAD-16–Aliquat 336 is shown in Figure 7. The best sorption of precious metal ions was observed from 0.1 M hydrochloric acid solutions. The graph clearly shows that, with an increase in the concentration of hydrochloric acid in the model noble metal solution, the ability of impregnated sorbent to sorb the noble metal ions decreases simultaneously. This is due to the larger content of chloride ions, which compete with the formed noble metal complexes and occupy functional groups on the sorbent surface. The best recovery was observed for gold(III) ions, when the 0.1 M HCl was 99.9%; at the 6 M HCl solution, it decreased slightly to 93.2%. The efficiency of platinum recovery was also high—in the 0.1 M HCl solution it was 99.99%, while in the 6 M HCl solution it equaled 86%. However, the sorption of palladium(II) ions was the weakest. In the 0.1 M HCl solution it was great—98.9%—but in higher concentrations of the HCl it dropped sharply; at the 6 M HCl solution, it was only 29%. At high HCl concentration, these metals occur in the form of stable chloro-complexes. Overall, the chloro-complexes of noble metals attach to functional groups on the sorbent surface in the order of MCl_6_^2−^ > MCl_4_^2−^ > MCl_6_^3−^ > aqua-complexes. This may be caused by the size, charge density and geometry of the chloro-complexes formed of these metals [25]. Gold(III) is the best sorbed element because its [AuCl_4_]^−^ complex has lower charge density than that of the other investigated elements, so its hydration shell is smaller (ion exchange occurs more readily). Platinum(IV) forms MCl_6_^2−^ complexes so it attaches more easily to the functional group of the sorbent than palladium(II) which forms MCl_4_^2−^.

#### 3.2.3. Influence of Contact Time

**Figure 8:**
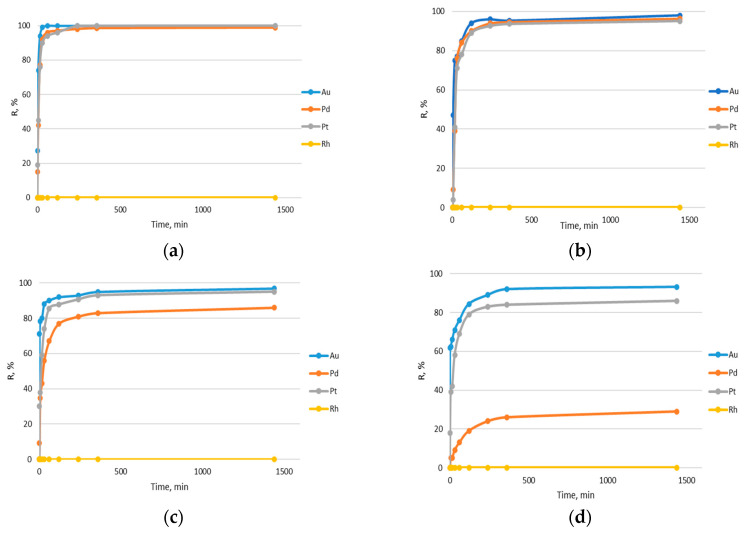
Dependence of removal percentage %R of noble metal ions on phase contact time at different concentrations of hydrochloric acid: (**a**) 0.1 M; (**b**) 1 M; (**c**) 3 M; (**d**) 6 M (each metal concentration: 10 ppm; sorbent mass: 0.2 g; solution volume: 50 mL).

To study the effect of contact time (1, 5, 15, 30, 60, 120, 240, 360, 1440 min) on noble metal sorption, 0.2 g of Amberlite XAD-16 was equilibrated with 50 mL of noble metal (Au(III), Pd(II), Pd(IV) and Rh(III)) solutions in the HCl concentration range: 0.1–6 M. The removal percentage was calculated from the equation:

(1) $$\%R=\frac{C}{C_{0}}\times 100\%$$

where %R is the removal percentage of the investigated noble metal ions, C is the concentration of the sorbed noble metal ions and C_0_ is the initial concentration of noble metal ions. Dependence of removal percentage of noble metal ions on phase contact time is presented in Figure 8. Rhodium(III) ions were not sorbed at all. For each of the hydrochloric acid solution concentrations, the amount of sorbed Au(III), Pt(IV) and Pt(II) ions increased with the phase contact time. Their sorption proceeded very quickly; in the case of 0.1 M HCl solution, more than 90% of all ions were removed after 15 min (Au(III)—99%, Pt(IV)—77%, Pd(II)—76%). With an increased concentration of chloride ions, the time to establish adsorption equilibrium increased. Thus, fewer metal ions were removed from solutions with higher acid concentrations due to competition between chloride ions and gold(III), platinum(IV) and palladium(II) ions.

### 3.3. Desorption Studies

**Figure 9:**
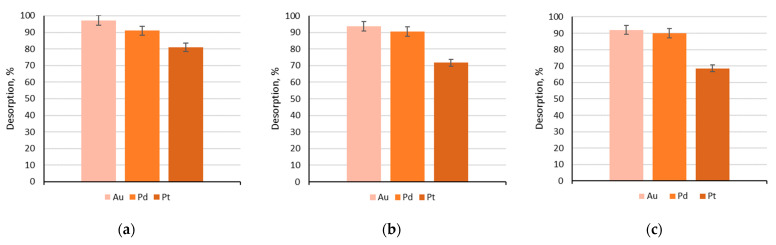
Desorption results from the impregnated sorbent Amberlite XAD-16–Aliquat 336 (sorbent mass: 0.2 g) using desorbing solutions: (**a**) 1 M thiourea + 1 M hydrochloric acid, (**b**) 0.5 M thiourea + 0.5 M hydrochloric acid, (**c**) 0.1 M thiourea + 0.1 M hydrochloric acid.

Desorption of sorbed Au(III), Pd(II) and Pt(IV) ions proceeded using three desorbing solutions: 1 M thiourea + 1 M hydrochloric acid solution, 0.5 M thiourea + 0.5 M hydrochloric acid solution and 0.1 M thiourea + 0.1 M hydrochloric acid solution. According to the literature data, thiourea in hydrochloric acid solution is frequently applied for the desorption of noble metal ions [26]. The results of research on Au(III), Pd(II) and Pt(IV) are presented in Figure 9.

The experiment proved that using each of the desorbing solutions, satisfactory results were obtained. The best gold(III), platinum(IV) and palladium desorption results were obtained for a solution containing 1 M thiourea and 1 M hydrochloric acid. The second highest desorption rate was obtained for the solution containing 0.5 M thiourea and 0.5 M hydrochloric acid. However, the poorest results among the tested systems were observed for the solution containing 0.1 M thiourea and 0.1 M hydrochloric acid. Desorption of rhodium(III) ions was not studied because ions of this metal do not sorb on Amberlite XAD-16–Aliquat 336.

### 3.4. Kinetic, Thermodynamic and Isotherm Studies

#### 3.4.1. Kinetics

By studying sorption kinetics, a likely adsorption pathway and dominant mechanism of the process can be determined. To study kinetics of gold(III), palladium(II) and platinum(IV) ions sorption on Amberlite XAD-16–Aliquat 336, experimental results were compared with two adsorption kinetic models (pseudo-first-order and pseudo-second-order) and two diffusion kinetic models (intra-particle diffusion model and Dumwald–Wagner model), which can be represented by the equations:

$$\ln\left(q_{e}-q_{t}\right)=lnq_{e}-k_{1}\times t\;(\mathrm{PFO})$$

$$\frac{t}{q_{t}}=\frac{1}{k_{2}\cdot q_{e}^{2}}+\frac{1}{q_{e}}\times t\;(\mathrm{PSO})$$

$$q_{t}=k_{ip}\times t^{0.5}+C\;(\mathrm{intra}\text{-}\mathrm{particle}\;\mathrm{diffusion})$$

$$\log\left(1-{\left(\frac{q_{t}}{q_{e}}\right)}^{2}\right)=-\frac{k}{2.303}t\;(\mathrm{Dumwald}\text{–}\mathrm{Wagner})$$

Kinetic parameters were calculated from the above equations and are presented in Table 1.

Based on the results presented in Table 1, it can be concluded that PSO is the kinetic model describing the best sorption of noble metals on the impregnated sorbent. The linear regression correlation coefficient (R^2^) for this model is above 0.999. The value of sorption capacity calculated using the PSO model was the closest to that determined from the experimental studies. The pseudo-second-order kinetic model assumes that chemical sorption is the process-controlling mechanism. Based on the equation of the intra-particle diffusion model, a graph of q_t_ from t^1/2^ was plotted and it was found to be not linear. It was evident that intra-particle diffusion is not the main mechanism occurring during the sorption of noble metal ions on Amberlite XAD-16–Aliquat 336. Moreover, the plot of log(1-F^2^) from t based on the Dumwald–Wagner equation was not linear with zero intercept, so diffusion through the liquid film covering the solid is not the governing mechanism of noble metal ions sorption on the impregnated sorbent. The literature reports indicate that the PSO model describes well the sorption of noble metals on modified sorbents [26,27].

#### 3.4.2. Thermodynamic Studies

A decision was made to study the effect of temperature on the sorption of noble metals on the impregnated sorbent for an accurate description of the process. Determining the thermodynamic parameters, it can be established whether sorption is chemical or physical in nature. Chemical adsorption is characterized by the presence of chemical bonds, while physical adsorption indicates the presence of weaker intermolecular interactions [28]. Most commonly, there are used three thermodynamic parameters to describe adsorption: Gibbs energy change (ΔG°), enthalpy change (ΔH°) and entropy change (ΔS°).

Gibbs free energy change can be calculated from the equation:

$$\Delta G{}^{\circ}=-RT\;ln\;K_{D}$$

where R is the universal gas constant (8.314 J/mol × K), T is the temperature (K) and K_D_ is the equilibrium constant. The equilibrium constant should be dimensionless, otherwise calculated parameters will be erroneous, as proved in some papers [29]. The Gibbs free energy change indicates sorption process spontaneity. The higher the value of this parameter, the more energetically favorable and spontaneous the process. The Gibbs free energy is related to entropy ΔS° and enthalpy change ΔH° based on the equation:

$$ln(K_{D})=-\frac{\Delta H{}^{\circ}}{RT}+\frac{\Delta S{}^{\circ}}{R}$$

Entropy ΔS° value provides information about the organization of the adsorbate at the solid/liquid interface. Positive values of this parameter suggest greater randomness in the organization of the adsorbate near the surface. In turn, the enthalpy change, ΔH°, indicates whether the sorption process is exothermic (ΔH° < 0) or endothermic (ΔH° > 0).

A graph of lnK_D_ vs 1/T was drawn to calculate the values of ΔH° and ΔS° from the slope and intercept. All calculated parameters are presented in Table 2.

As mentioned earlier, Gibbs free energy change is responsible for the spontaneity and feasibility of the process. Its value was negative for each element, thus indicating that sorption occurred favourably and spontaneously. Positive values of entropy show that accumulation of adsorbate at the interface is more random than organized. Moreover, it can be concluded that the process is endothermic, as this is indicated by the enthalpy change, which is positive for each metal.

#### 3.4.3. Isotherms

For reliable descriptions of interactions between adsorbate and adsorbent, adsorption isotherm models are used. Thus, it is possible to describe adsorption phenomenon, the capacity of the adsorbents and a probable path of the process. The isotherm equations used to describe the sorption of Au(III), Pt(IV) and Pd(II) ions are shown below:

$$q_{e}=\frac{q_{m}K_{L}C_{e}}{1+K_{L}C_{e}}\;(\mathrm{Langmuir})$$

where q_e_ is the amount of adsorbate (mg/g), q_m_ is the maximum adsorption capacity (mg/g), K_L_ is the constant related to the adsorption capacity and C_e_ is the equilibrium concentration in the solution.

The dimensionless constant R_L_ is defined as follows:

$$R_{L}=\frac{1}{1+K_{L}C_{e}}$$

As follows, if adsorption is favourable, its value is 0–1:

$$q_{e}=K_{F}{C_{e}}^{\frac{1}{n}}\;(\mathrm{Freundlich})$$

where q_e_ is the amount of adsorbate (mg/g), K_F_ is the constant related to the adsorption capacity (L/g), C_e_ is the equilibrium concentration in the solution (mg/L) and n is the adsorption intensity.

$$\frac{q_{e}}{q_{m}}=\frac{RT}{b}lnK_{T}C_{e}\;(\mathrm{Temkin})$$

where q_e_ is the amount of adsorbate (mg/g), q_m_ is the maximum adsorption capacity (mg/g), K_T_ is the constant related to the adsorption capacity, C_e_ is the equilibrium concentration in the solution, R is the universal gas constant −8.314 J/K × mol and T is the temperature.

The results calculated from the above isotherm equations are presented in Table 3.

Based on the results, the Langmuir isotherm model proved to be the best fitted isotherm for Au(III), Pt(IV) and Pd(II) ions sorption. According to the Langmuir theory, each site on the surface of a sorbent can sorb only one molecule, resulting in an adsorbate forming a monolayer on the adsorbent surface, which is also a reversible process. The determination coefficients for Au(III), Pt(IV) and Pd(II) ions were 0.9955, 0.9834 and 0.9997, respectively. The sorption capacities determined from the calculations for this model (Au(III)—99.12 mg/g; Pt(IV)—46.69 mg/g; Pd(II)—46.26 mg/g) were the closest to the experimental ones: 94.34 mg/g for Au(III) ions, 45.35 for Pt(IV) ions and 46.03 mg/g for Pd(II) ions. Taking into account the R_L_ factor, sorption of the investigated noble metal ions is favourable. The obtained sorption capacity on Amberlite XAD 16–Aliquat 336 are high compared with the literature data. The maximum Au(III) adsorption capacity on XAD7 resin modified with L-glutamic acid was 14.23 mg/g [27]. In turn, palladium(II) was sorbed onto Kieselguhr immobilized with diphenylthiocarbazone sorption capacity up to 11.2 mg/g [26].

### 3.5. Noble Metal Recovery from the Spent RAM Module

**Figure 10:**
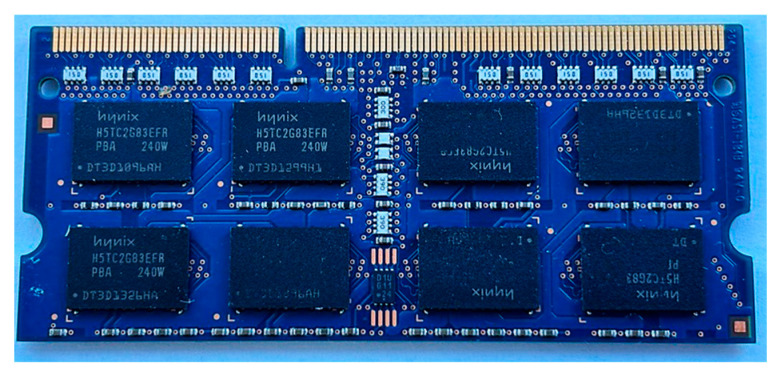
Studied spent RAM module.

A spent random access memory module dismantled from a computer (Figure 10) was treated with leaching solution: 36% hydrochloric acid + 30% hydrogen peroxide. The RAM module weighed 7.8040 g. In Table 4, the concentrations of metal ions in the solution after leaching are presented.

**Figure 11:**
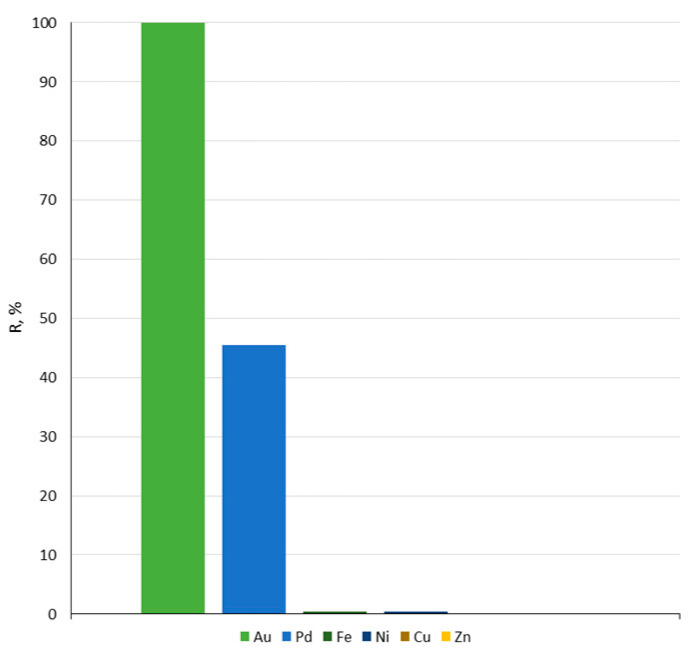
Percentage of metal ions recovery from the RAM module real leaching solution (solution volume: 25 mL; sorbent mass: 0.1 g).

Based on the values shown in Table 4, it can be concluded that the main elements that the RAM module is composed of are primarily copper, as well as nickel. A total of 25 mL of the doubly diluted solution was obtained after the RAM plate digestion was contacted with 0.1 g of Amberlite XAD-16–Aliquat 336 impregnated sorbent. Selectivity of the sorbent towards gold(III) and palladium(II) ions was studied. Below, Figure 11 shows the results of sorption on Amberlite XAD-16–Aliquat 336 from a real solution containing noble metals. The efficiency of gold(III) ions sorption was 99.9%. Only 45.4% of palladium(II) ions were sorbed. The priority in sorption of gold(III) complexes is explained in Section 3.2.2. The remaining metal ions were not sorbed by the sorbent. The results clearly indicate that Amberlite XAD-16 impregnated with Aliquat 336 is selective towards Au(III) ions.

After sorption of noble metal ions from the leaching solution, desorption studies were carried out. A total of 0.5 M thiourea + 0.5 M hydrochloric acid solution was used for the desorption of gold(III) and palladium(II) ions. The desorption (%D) of gold(III) was 99.84% while that of palladium(II) ions was 91.6%.

## 4. Conclusions

Polymeric resin Amberlite XAD-16 was impregnated using a novel “warm impregnation” method using quaternary ammonium salt Aliquat 336. The obtained impregnated sorbent was characterized by FTIR-ATR, SEM amd ASAP analysis. The suitability of the synthesized resin for the sorption of noble metals such as Au(III), Pt(IV), Pd(II) and Rh(III) was investigated. The effect of various factors (HCl acid concentration, phase contact time, sorbent mass) on the sorption of these metals was studied. It was observed that rhodium(III) ions were not sorbed on Amberlite XAD-16–Aliquat 336. The other noble metals were effectively sorbed by the prepared sorbent. The optimum sorbent mass in the further study proved to be 0.1 g. The best sorption was found from 0.1 M hydrochloric acid solution and the best sorbed element was Au(III). At a high concentration of HCl, sorption decreased due to the competition between chloride ions and noble metal ions. The metal ions desorbed from the sorbent using a solution containing 1 M thiourea and 1 M hydrochloric acid. The best-fitted kinetic model was the pseudo-second-order model which indicates that the sorption of noble metal ions followed a chemisorption mechanism. In turn, the best adsorption isotherm model to describe this process was the Langmuir isotherm one. The determination coefficient for this model for Au(III) was 0.9955, for Pt(IV) was 0.9834 and for Pd(II) was 0.9997. The experimental sorption capacities for each metal were: Au(III)—94.34 mg/g, Pt(IV)—45.35 mg/g and Pd(II)—46.03 mg/g. The RAM module from the spent computer was digested in the solution containing 36% hydrochloric acid, 30% hydrogen peroxide and water. Using Amberlite XAD-16–Aliquat 336, 99.9% of the gold(III) ions and 45.4% of the palladium(II) ions were removed from the real leaching solution. The above results confirm the applicability of the newly synthesized Amberlite XAD-16 sorbent impregnated with Aliquat 336 for the removal of noble metal ions from model solutions as well as from real solutions obtained after e-waste leaching.

## Figures and Tables

**Table 1:** Kinetic parameters for Au(III), Pd(II) and Pt(IV) ions.

**HCl** **Concentration**	**Noble Metal Ion**	**q_m_ (mg/g)**	**Pseudo-First-Order**			**Pseudo-Second-Order**		
			**k_1_ (L/min)**	**q_e_ (mg/g)**	**R^2^**	**k_2_ (L/min)**	**q_e_ (mg/g)**	**R^2^**
0.1 M	Au(III)	2.50	0.00949	0.1882	0.4450	0.32016	2.4966	0.9999
	Pt(IV)	2.50	0.01833	0.8504	0.9297	0.07888	2.5346	0.9999
	Pd(II)	2.47	0.01371	0.5722	0.7856	0.56958	2.4965	0.9998
1 M	Au(III)	3.74	0.00766	0.5280	0.7400	0.14691	3.7005	0.9998
	Pt(IV)	2.65	0.01112	0.8091	0.8179	0.06773	2.6644	0.9999
	Pd(II)	2.48	0.00185	2.1225	0.4303	0.06829	2.4934	0.9998
3 M	Au(III)	2.42	0.00620	0.3832	0.8409	0.18192	2.3738	0.9998
	Pt(IV)	2.38	0.00886	0.8902	0.8445	0.06112	2.3554	0.9996
	Pd(II)	2.15	0.00456	1.3444	0. 7828	0.03739	2.1372	0.9993
6 M	Au(III)	2.33	0.00887	0.7576	0.9923	0.06321	2.3143	0.9987
	Pt(IV)	2.40	0.00969	1.0379	0.9102	0.04672	2.4032	0.9991
	Pd(II)	0.78	0.00089	2.1443	0.8658	0.02970	0.7692	0.9864
**HCl** **Concentration**	**Noble Metal Ion**	**Dumwald–Wagner**			**Intra-Particle Diffusion**			
		**K (min^−1^)**		**R^2^**	**K (m/g × min^0.5^)**		**C (mg/g)**	**R^2^**
0.1 M	Au(III)	0.00897		0.4620	0.0245		2.4998	0.2214
	Pt(IV)	0.01749		0.9471	0.0379		2.4998	0.3684
	Pd(II)	0.01285		0.8212	0.0377		2.4700	0.3316
1 M	Au(III)	0.00515		0.4845	0.0242		3.7425	0.4367
	Pt(IV)	0.01020		0.8581	0.0416		2.6500	0.3775
	Pd(II)	0.00116		0.5004	0.0401		2.4825	0.3613
3 M	Au(III)	0.00415		0.6296	0.0145		2.4225	0.5973
	Pt(IV)	0.00801		0.8787	0.0377		2.3750	0.5056
	Pd(II)	0.00355		0.8451	0.0425		2.1500	0.5645
6 M	Au(III)	0.00519		0.9232	0.0233		2.3300	0.7254
	Pt(IV)	0.00881		0.9323	0.0395		2.4000	0.5683
	Pd(II)	0.00027		0.9538	0.0201		0.7750	0.7708

**Table 2:** Thermodynamic parameters for Au(III), Pd(II) and Pt(IV) ions.

Parameter		Au(III)	Pt(IV)	Pd(II)
ΔG°	T = 293 K	−9.15	−4.29	−6.92
	T = 303 K	−11.69	−4.48	−11.3
	T = 333 K	−12.98	−4.95	−12.98
ΔS°		192.85	32.28	305.89
ΔH°		47.16	5.21	82.34

**Table 3:** Freundlich, Langmuir and Temkin isotherm model parameters for Au(III), Pd(II) and Pt(IV) ions.

Isotherm Model	Parameter	Au(III)	Pt(IV)	Pd(II)
Freundlich	K_F_	25.65	18.05	11.75
	n	4.65	3.42	3.59
	R^2^	0.8581	0.8809	0.6443
Langmuir	Q_0_	99.12	46.69	46.26
	K_L_	0.1639	0.064	0.3911
	R_L_	0.1162	0.2136	0.0584
	R^2^	0.9955	0.9834	0.9997
Temkin	K_T_	3.27	6.86	162.03
	b_T_	1.53	1.83	12.43
	R^2^	0.8161	0.4296	0.8231

**Table 4:** Metal contents in the real leaching solution.

Metal Ions	Fe^3+^	Ni^2+^	Cu^2+^	Zn^2+^	Pd^2+^	Au^3+^
Concentration (mg/L)	4.54	490	7018	1.12	1.76	38.10

## Data Availability

Data are contained within the article.
